# Study on Rapid Detection Method for Degradation Performance of Polyolefin-Based Degradable Plastics

**DOI:** 10.3390/polym15010183

**Published:** 2022-12-30

**Authors:** Jinglun Zhou, Linlin Li, Dengxu Wang, Lihong Wang, Yuanqi Zhang, Shengyu Feng

**Affiliations:** 1 Key Laboratory of Special Functional Aggregated Materials, Ministry of Education, School of Chemistry and Chemical Engineering, Shandong University, Jinan 250100, China; 2 Shandong Institute for Product Quality Inspection, Jinan 250100, China; 3 Eco-Benign Plastics Technology Company Limited, Jinan 250101, China

**Keywords:** bioassimilated carbon, polyolefin-based degradable plastics, detection, degradation performance

## Abstract

In order to accurately determine the degradation performance of polyolefin-based degradable plastics, the concept of bioassimilated carbon is proposed for the first time in this paper; the bioactive and hydrophilic organic carbon in plastic degradation products is defined as bioassimilation carbon. A method for the detection of the carbonyl index and bioassimilated carbon conversion rate in polyolefin degradable plastics was developed to quickly identify its degradation performance. The measurement results show that the bioassimilated carbon conversion rate of more than 70% can be used to replace the biodegradation rate index to achieve the purpose of quickly identifying the degradation performance of plastics. The deterioration detection cycle proposed by the current common standards implemented in American Society of Testing Materials: ASTM D6400 “Specification for Composting Plastics” can be shortened from 1 year to 1 month. The standard system for catalytic degradation of plastics provides detection methods for polyolefin-based catalytic degradation materials (microplastics), and solves the problems of long detection cycle and poor detection efficiency. Thus, this method has promise for use as a relevant standard method for accurately providing a reference for the assessment.

## 1. Introduction

Since the implementation of plastic restriction policies, a variety of degradable plastics have flourished [1,2]. Compared with traditional non-degradable materials, these green materials have attracted much attention due to their biodegradable properties [3,4,5,6]. The degradable plastics can be classified into compostable degradable ones and environmentally degradable ones according to the source of raw materials and their degradation properties [7,8,9,10]. The former mainly includes polylactic acid (PLA), poly(butyleneadipate-co-terephthalate) (PBAT), modified starch, polybutylene succinate (PBS), etc. [11,12,13,14,15,16,17]. The main materials that can be decomposed in the natural environment are made by compounding polyolefin materials, such as polyethylene (PE), polypropylene (PP) and degrading additives. These materials have many advantages such as superior physical properties and low cost. In addition, on-demand degradable materials can also be prepared through different degradable additives, especially in the application of degradable PE-based mulch films, which are favored by consumers such as farmers and facility vegetable industries, and thus have broad market prospects [18,19,20,21]. However, the appearance and performance of the above-mentioned degradable materials before degradation are almost the same as those of traditional PP or PE materials without degradable additives [22], resulting in a mixed bag of such products on the market, resulting in regulatory difficulties.

At present, the standard methods for detecting the degradation performance of polyolefin-based degradable plastics are mainly based on the rates of the preserved elongation at break and the decreased molecular weight [23,24]. The percentage of molecules with an average molecular weight of less than 10,000 g·mol^−1^ can be tested by these methods. Although the molecular weight and mechanical strength of the product can be reduced, it cannot be determined whether it can be further biodegraded by microorganisms. For example, the degradation of a type of polyolefin and inorganic salt composite products cannot be determined under this method, although they meet the above requirements after degradation. However, its structure and properties are still plastic and it may also cause serious environmental and health hazards to humans due to the formation of microplastics [25,26]. In ASTM6954 promulgated by American Society of Testing Materialss and PAS9017:2020 published by the Publicly Available Specification [27,28], in addition to various molecular weights, the index of carbonyl index and biodegradation rate were also increased. For example, the degree of oxidation of these materials can be verified by evaluating the carbonyl index, and the biodegradation rate can demonstrate whether the material is ultimately decomposable into carbon dioxide and water. These properties can reveal the existence of microplastics and avoid the secondary harm of microplastics to the environment. However, the carbonyl index is usually monitored by infrared spectroscopy. Most of the infrared spectra of products are disorganized, which can easily lead to inaccurate integration and large analysis errors, resulting in poor reproducibility of test results in different laboratories. Whether it is in soil or in compost, in order to achieve the biodegradation rate of more than 90%, the test time should be at least 6 months [29,30], and the deterioration detection period proposed by the existing standard is 1 year. The testing cost of a single experimental group is about 10,000–30,000 yuan. In recent years, the explosive growth of the degradable plastics industry has led to a rapid increase in the demand for testing. However, such problems as long testing cycles, high costs, and inadequate industry supervision have seriously restricted the rapid development of the industry. In order to meet the growing demand for detection and identification and promote the healthy and sustainable development of the industry, it is imperative to innovate and formulate methods for rapid detection and identification of the degradation properties of polyolefin-based degradable plastics. The real degradation behavior of polyolefin plastics in the natural environment is that they can be further broken down by microorganisms. The premise is that inert organic carbon can be oxidized to bioassimilated carbon.

In the present study, we propose and optimize a new method to test the degradation performance of polyolefin degradable plastics by measuring the conversion rate of bioassimilated carbon, which can shorten the degradation performance detection period to 1 month. The results are simple and accurate, and provide a research basis for research and development, modification optimization and a rapid detection standard system of polyolefin degradable plastics.

## 2. Materials and Methods

### 2.1. Materials and Characterization 

Polyolefin-based degradable plastic and PP rigid sheet were provided by Shandong Tianzhuang Environmental Protection Technology Co., Ltd. (Jinan, China). The organic carbon was analyzed by elementary Acquray TOC (total organic carbon analyzer). The Xenon lamp aging environmental test chamber was analyzed by Q-Sun Xe-1. The molecular weight was measured by Agilent GPC-220 high temperature gel chromatography. Fourier transform infrared spectra were measured via a KBr pellet technique within a 4000 to 400 cm^−1^ region on a Bruker TENSOR-27 infrared spectrophotometer. 

### 2.2. Aging Test of Polyolefin-Based Plastics

The samples were cut into 5 cm× 20 cm rectangles. According to the requirements of the GB/T16422.2 standard, the black mark temperature is 65 °C ± 0.5 °C. Relative humidity is 65 ± 0.5%. The test samples are sprayed with water every 102 ± 0.5 min for 18 min ± 0.5 min in a simulated rainy day environment. The total radiation dose is 26 MJ/m^2^. The degradable polyolefin plastic film and rigid sheet are tested under the laboratory xenon lamp aging conditions that can simulate natural environmental aging.

The samples with the weight of 0.02 g to 0.04 g (accurate to 0.01 mg) before and after degradation were put into 300 mL conical flasks. 10 mL of potassium dichromate standard solution (0.8 mol/L) and 10 mL of sulfuric acid solution (18.4 mol/L) were added. The curved neck funnel was then attached to the flask. The apparatus was placed in a boiling water bath for 45 min. After cooling to room temperature, 80 mL of water and 2–3 drops of phenanthroline indicator were added. Then ferrous sulfate standard titration solution was used to titrate the resultant solution to the end point by monitoring the color of the solution changing from green to dark green to brick red. Meanwhile, 0.1 g of silica was used instead of the sample as a comparative experiment.

The degradation performance of degradable plastics is expressed by the conversion rate of bioassimilated carbon, and the calculation method is expressed as Equation (1) [31].

W(%) = *X/X*_0_ × 100 (1)

where W is the mass fraction (%) of the conversion rate of biological assimilation carbon; *X* is the mass fraction of bioassimilated carbon (%); *X*_0_ is the mass fraction (%) of total organic carbon in the sample before degradation.

The mass fraction *X* (%) of bioassimilated carbon is calculated using the following Equation (2) [31].

(2) $$X(\%)=\frac{C\times (V_{0}-V)\times 3}{m\times 1000}\times 100$$

where *X* is the mass fraction of bioassimilated carbon in the sample (%); *C* is the concentration of ferrous sulfate standard solution (mol/L); *V* and *V*_0_ are the volumes of ferrous sulfate standard solution consumed by the sample and blank solution, respectively (mL); *m* is the total mass of carbon in the sample (g); 3 is the numerical value (g/mol) of the molar mass of a quarter of a carbon atom.

## 3. Results and Discussion

### 3.1. Degradation Mechanism of Degradable Plastics 

**Figure 1:**
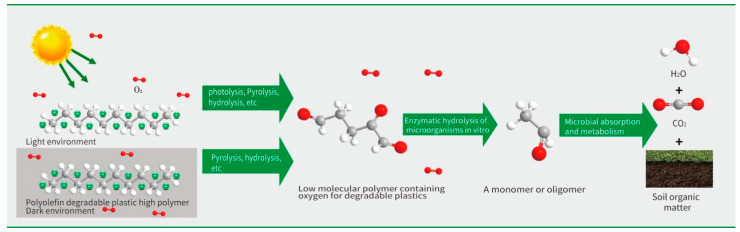
Degradation process of degradable plastic products.

The breakage of polymer chemical bonds is the essence of biodegradable plastics degradation. The degradation process of polyolefin biodegradable plastics can be divided into chemical degradation and biodegradation. The chemical degradation under the conditions of light, heat, radiation and mechanical stress entails the polymer reacting with oxygen to generate a large number of oxidation products, such as aldehydes, ketones and acids. At the same time, the separation of the active test group from the main chain leads to the change of the main chain structure, and the main chain breaks, becoming a low molecular weight polymer. On the whole, the biodegradation process of plastic is generally a transformation from the original long polymer chains into polymer chain segments with low molecular weight through hydrolysis and thermal oxygen degradation, and then decomposition into monomers or oligomers by hydrolase secreted by microorganisms in vitro. Then the oligomers or monomers are hydrolyzed into CO_2_, H_2_O, CH_4_, and other small molecules through microbial absorption and metabolic reactions. The process is shown in Figure 1. Polymers depolymerize through hydrolysis, thermal oxygen and other actions, and the process of producing oligomers and monomers is the rate-limiting step of biodegradable plastics degradation. Therefore, different catalytic materials are mixed in the production, and the introduction of weak chemical bonds or reactive chemical bonds can make the products more prone to degradation. Based on this degradation mechanism, herein a method is presented to verify the degradation performance and degradation rate of target samples by measuring the carbon conversion rate of bioassimilated carbon, the reduction rate of molecular weight of samples, and the carbonyl index during biodegradation.

### 3.2. Screening of Oxidants and Determination of Oxidation Time 

This volumetric analysis method belongs to the redox reaction. In this study, the redox system is divided into two combinations. The strong oxidant potassium dichromate (K_2_Cr_2_O_7_) or potassium permanganate (KMnO_4_) under acidic conditions is used to measure the reducing properties of polyolefin-based plastics. For bioactive carbon oxidation, titrating the remaining solution with ferrous sulfate (FeSO_4_) or oxalic acid (H_2_C_2_O_4_), we can indirectly calculate the bioassimilated carbon content in the polyolefin material after environmental degradation by calculating the volume of ferrous sulfate or oxalic acid consumed. Group 1 uses a combination of strong oxidants and ferrous sulfate for the oxidation of bioactive carbon in polyolefin plastics under acidic conditions, and the experimental results are summarized in Table 1. The smaller the relative standard deviation, the higher the accuracy. The results show that the bioassimilated carbon content of the detection reaction system based on potassium dichromate/ferrous sulfate is generally higher than that of the potassium permanganate/oxalic acid system. Therefore, the potassium dichromate/ferrous sulfate system was preferentially selected as the redox reaction system tested.

Using potassium dichromate/ferrous sulfate as the redox system, the release content of bioassimilated carbon at different reaction times was determined, and the optimal oxidation time was screened. As shown in Table 2, in the potassium dichromate/ferrous sulfate redox system, the results of the same polyolefin degradation products showed an increasing trend from 15 min to 45 min during the reaction process. The results stabilized when the reaction time was from 45 min to 1 h. Comparing the results of 15 min, 30 min, 45 min and 60 min in Table 2, 45 min was selected as the optimal reaction time.

### 3.3. Analysis of Bioassimilated Carbon Release under Different Aging Times

**Figure 2:**
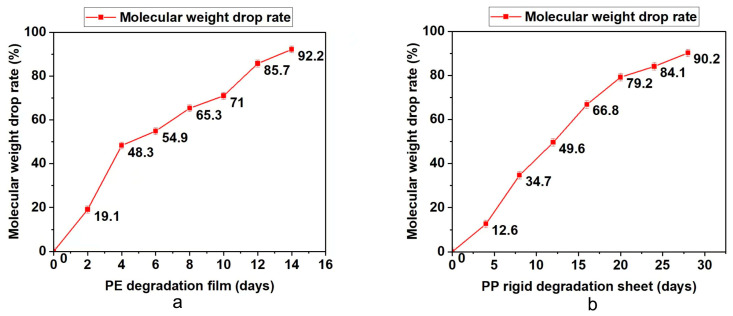
(**a**) Determination of molecular weight of PE during degradation. (**b**) Determination of molecular weight of PP during degradation.

**Figure 3:**
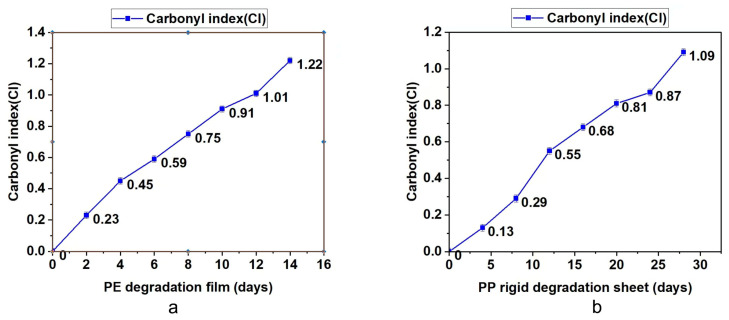
(**a**) Determination of carbonyl index during degradation of PE materials. (**b**) Determination of carbonyl index during degradation of PP materials.

**Figure 4:**
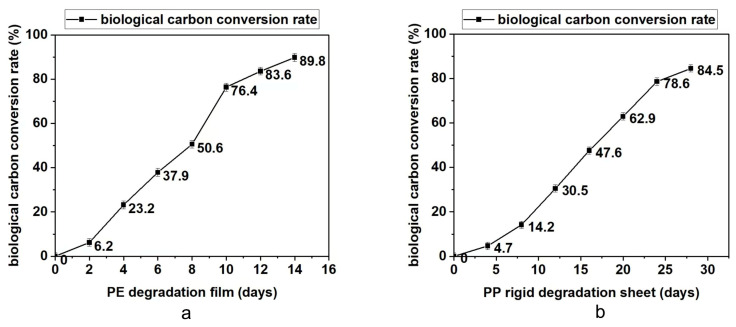
(**a**) Determination of bioassimilated carbon conversion during degradation of PE materials. (**b**) Determination of bioassimilated carbon conversion during degradation of PP materials.

The degradable polyolefin plastic film and rigid sheet are tested under the laboratory xenon lamp aging conditions that can simulate natural environment aging. According to British Standard PF 9017-2021, the test periods for PE film and PP rigid sheet are 14 days and 28 days. Every quarter of the test cycle, part of the material was taken out to test the molecular weight, infrared and bioassimilated carbon conversion. The results are summarized in Table 3 and Figure 2, Figure 3 and Figure 4. On the basis of these results, it was found that with the prolongation of aging time, the molecular weight drop rate, carbonyl index and bioassimilated carbon conversion rate of degradation products have a positive relationship. The conversion rates of bioassimilated carbon after 14 days of degradation of olefin film samples and 28 days of PP hard materials were 89.8% and 84.5%, the carbonyl index was 1.22 and 1.09, and the molecular weight drop rate of degradation products was 92.2% and 90.2%, respectively. The results are in line with the existing general detection standards.

### 3.4. Comparative of Bioassimilated Carbon Content and Biodegradation Rate under the Same Aging Conditions

In order to verify the consistency of the test results of bioassimilated carbon conversion rate and biodegradation rate, the biodegradation rate test was carried out on the polyolefin degradation products (degradation products of PE film after 14 days of degradation treatment) according to the GB/T19277.1-2011 “Determination of Final Aerobic Biodecomposition Capacity of Materials under Controlled Compost Conditions—by means of Determination of released carbon dioxide” test method. On day 173, the biodegradation rate of this material has reached more than 90%. The results prove that the polyolefin degradable material oxidized to a certain degree can meet the test index requirements of the biodegradation rate of bio-based fully biodegradable materials. The specific test data and the amount of carbon dioxide released and biodegradation rates are summarized in Table 4. The average carbon dioxide release amount of the test sample (degradation products of PE film) in 173 days is 167.56 g; the average carbon dioxide release amount of plant cellulose is 120.15 g; the average carbon dioxide release amount of the blank control (activated vermiculite) is 42.81 g. It is proved that the biodecomposition performance of polyolefin degradable materials can be well demonstrated by measuring the conversion rate of bioassimilated carbon to achieve the purpose of rapid identification. The experimental effect is consistent with GB/T19277.1-2011 [30].

### 3.5. Comparative of Different Detection Methods

By comparing the current common detection methods [32], as shown in Table 5, the results show that nuclear magnetic resonance spectroscopy can test the degradation performance of degradable plastics by qualitative and quantitative detection of the composition of degradable plastics, which is suitable for the whole biodegradation of plastic products made with PBAT, PBS, PLA, starch as the main raw materials. However, the detection cost is high on the basis of the expensive equipment required and the method is low in popularity. Infrared spectroscopy/Raman spectroscopy, differential scanning calorimetry and thermogravimetric analysis are relatively simple and fast to operate [33]. Using IR/Raman spectroscopy, the infrared spectrum characteristic absorption peaks of different materials for qualitative identification and the material composition were measured. However, this method is not applicable to the black plastic products [34], and needs a variety of methods to identify products’ composition; the test steps are complex, there is a big error, and related technical standards have not yet fully established, with a low value for reference. At present, the commonly used testing standard mainly adopts the compost fermentation method, and the testing cycle is 150–180 days [30]. The testing cycle is long and the efficiency is low. The above methods cannot meet the testing needs of enterprises, scientific research institutes, and regulatory authorities to develop new products and test market products. In contrast, the present biological carbon assimilation tests cost can be used for only 80–100 yuan/sample, and this test is suitable for all biodegradable plastic products, oxidation biological degradation plastics products and so on. Therefore, its application scope is wide. Moreover, the testing time can be reduced to 30 days, much shorter than other methods. In addition, the test operation is simple, convenient, can be powerfully generalized and meet the market demand for plastic degradation of performance evaluation.

## 4. Conclusions

This study reports the development of a rapid detection method for the degradation performance of degradable polyolefin plastics by testing the biodegradation rate of the degradation products in the composting environment. This method is the first application of this approach in the field of biodegradable plastics detection, and the test results are consistent with the test results of the general detection method GB/T 19277.1-2011 [30]. The bioassimilable carbon detection method can quickly reflect the degradation performance of biodegradable plastics. The conversion rate of bioassimilated carbon in different degradation stages was consistent with the decreased rate of molecular weight and the increased carbonyl index of the tested samples. This method can not only dynamically study the effectiveness of the degradation process of polyolefin materials, but also identify the biodegradability of the final product under certain aging conditions. More importantly, it can be calculated from Table 5 that compared with the current internationally recognized standard method GB/T 19277.1 [30], the testing cost of the new testing method can be reduced by more than 80%, and the detection period can be shortened to 1 month, and the sample pretreatment is simple. This method is fast, efficient and economical, and is more conducive to promotion and application in production enterprises, third-party testing laboratories, market supervision departments, etc.

## Figures and Tables

**Table 1:** Comparison of bioassimilated carbon content in two redox systems.

Test Group (%)	Redox System (%)	
	Potassium Dichromate/Ferrous Sulfate	Potassium Permanganate/Oxalic Acid
1	55.24	52.00
2	54.91	52.58
3	55.32	52.44
4	54.52	52.96
5	54.69	53.32
6	55.19	53.01
7	55.04	52.47
8	55.27	53.87
9	55.15	52.69
10	54.73	53.14
Relative standard deviation RSD%	0.66	1.51

**Table 2:** Comparison of bioassimilated carbon content at different reaction times in potassium dichromate/ferrous sulfate redox system.

Test Group (%)	Reaction Time (min)			
	15	30	45	60
1	36.55	43.66	55.24	55.34
2	31.42	41.78	54.91	55.03
3	33.78	42.63	55.32	55.35
4	37.65	45.61	54.52	54.67
5	32.89	42.54	54.69	54.78
6	30.25	40.68	55.19	55.35
7	33.49	41.59	54.04	55.09
8	34.97	44.32	55.27	55.19
9	36.01	46.01	55.15	55.29
10	31.86	41.00	54.73	54.90
Relative standard deviation, RSD%	9.79	4.38	0.66	0.56

**Table 3:** Test analysis of carbonyl index, molecular weight and bioassimilated carbon conversion rate of PE and PP materials.

Test Items	Time (d)	Molecular Weight Drop Rate/%	Carbonyl Index (CI)	Biological Carbon Conversion Rate
PE degradation film	0	0	0	0
	2	19.1	0.23	6.2
	4	48.3	0.45	23.2
	6	54.9	0.59	37.9
	8	65.3	0.75	50.6
	10	71.0	0.91	76.4
	12	85.7	1.01	83.6
	14	92.2	1.22	89.8
PP rigid degradation sheet	0	0	0	0
	4	12.6	0.13	4.7
	8	34.7	0.29	14.2
	12	49.6	0.55	30.5
	16	66.8	0.68	47.6
	20	79.2	0.81	62.9
	24	84.1	0.87	78.6
	28	90.2	1.09	84.5

**Table 4:** Carbon dioxide emission.

Item Category	Test Group	45 Days	173 Days
CO_2_ release in blank test/g	Activated vermiculite 1	15.35	43.71
	Activated vermiculite 2	14.47	41.10
	Activated vermiculite 3	13.91	43.63
CO_2_ release of test sample/(actual)g	Degradation products of PE film 1	73.78	166.73
	Degradation products of PE film 2	76.66	170.37
	Degradation products of PE film 3	77.11	165.58
CO_2_ release of reference material/(actual) g	Plant cellulose 1	82.88	120.23
	Plant cellulose 2	78.36	121.12
	Plant cellulose 3	78.91	119.11
CO_2_ emission of test samples/(theory) g	138.25		
CO_2_ emission of reference material/(theory) g	79.13		

**Table 5:** Comparison of different detection methods.

Methods	Detection Time/Day	Test Cost/Sample/USD	The Difficulty of Testing Technology	Scope of Application
Nuclear magnetic resonance spectroscopy	25–30	55–110	Medium operation difficulty, large equipment investment, need to entrust a professional testing company, the method has low popularity	Fully biodegradable plastic products
Differential scanning calorimetry	25–30	55–110	The operation is difficult, the procedure is tedious, the equipment investment is large, the utility of the method can be generalized is low	PLA based plastic products
Thermogravimetric analysis	30–40	55–110	The operation difficulty is medium, the procedure is tedious, the equipment investment is large, the possibility for generalization is low	Starch based plastic products
Infrared spectroscopy/Raman spectroscopy	30–50	40	Simple operation, large equipment investment, need to entrust a professional testing company, the method is low in popularity	All biodegradable plastics of any color other than black
Compost fermentation	180–360	2100	Low operation difficulty, low detection efficiency, large test error, low generalization of the method	Fully biodegradable plastic products
Natural degradation process	600	850	Low operation difficulty, low detection efficiency, large test error, low generalization of the method	Fully biodegradable plastic products
This work	15–30	55–70	Simple operation, equipment, reagents and other materials are easy to obtain, the method is easy to popularize	Biodegradable plastic products and oxidized—biodegradable plastic products

## Data Availability

All the data have been given in the manuscript.
